# Histiocytoid Sweet’s Syndrome in the Setting of Myelodysplastic Syndrome

**DOI:** 10.7759/cureus.59161

**Published:** 2024-04-27

**Authors:** Isha Jhingan, Chase A Pitchford, Michael B Franzetti, Jeffrey D McBride, Jarad I Levin

**Affiliations:** 1 Dermatology, University of Oklahoma Health Sciences Center, Oklahoma City, USA

**Keywords:** malignancy-associated sweet syndrome, clinical dermatology, myelodysplasia, sweet's syndrome, histiocytoid

## Abstract

Acute febrile neutrophilic dermatosis, or Sweet’s syndrome, is characterized by tender, edematous papules and plaques, favoring the upper extremities and the head and neck regions. The classic variant of Sweet’s syndrome involves a predominantly neutrophilic dermal infiltrate on histopathology. However, histiocytoid Sweet’s syndrome has been noted to have a primary histiocytoid mononuclear infiltrate and is typically found in patients with malignancies such as myelodysplasia. This case report discusses the treatment of histiocytoid Sweet’s syndrome in an immunocompromised patient with a recent history of *Mycobacterium avium* complex infection and latent tuberculosis in the setting of myelodysplastic syndrome.

## Introduction

Acute febrile neutrophilic dermatosis, or Sweet’s syndrome, is characterized by an eruption of tender, edematous papules and plaques, typically involving the upper extremities, head, and neck region [1]. Areas outside the skin, such as the eyes, can also be affected. Other clinical symptoms of the condition include fever and leukocytosis. Robert Douglas Sweet identified this condition in 1964, which was then subsequently named Sweet’s syndrome. Sweet’s syndrome is considered a reactive dermatologic condition with usual triggers including an infection or new medication exposure [1]. Another potential cause of the disorder can be both hematological cancers, namely, acute myelogenous leukemia, or solid organ neoplasms [2]. While Sweet’s syndrome is traditionally considered a neutrophilic dermatosis, histiocytoid Sweet’s syndrome is an unusual pathological presentation of Sweet’s syndrome with similar clinical features but differing histology [3]. Instead of finding a predominantly neutrophilic dermal infiltrate, histiocytoid Sweet’s syndrome is characterized by a histiocytic mononuclear infiltrate [3].

While the pathophysiology of Sweet’s syndrome is not clearly defined, a defect in the production and secretion of cytokines such as interleukin-1 and granulocyte colony-stimulating factor is the main hypothesis [1]. These immune system proteins become overstimulated and promote unnecessary neutrophil proliferation and migration to the skin, which ultimately results in tissue damage. This effect is then visualized in the histology of the neutrophilic variant of Sweet’s syndrome.

Typical treatment for Sweet’s syndrome includes systemic corticosteroids to help control the disease. Topical steroids may also be utilized in the setting of visual lesions. Dapsone, potassium iodide, and colchicine are recommended second-line therapies [1].

## Case presentation

**Figure 1:**
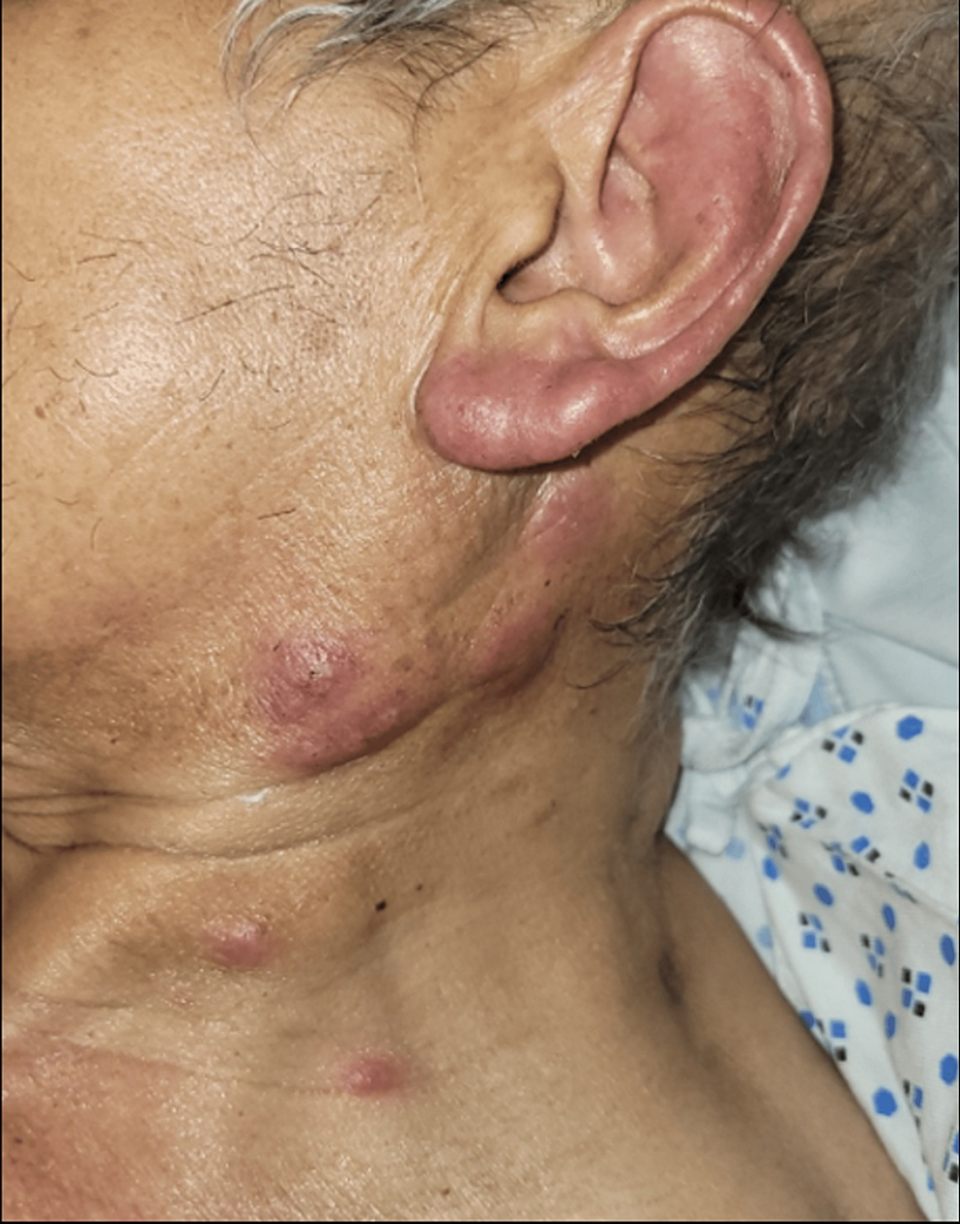
Juicy, edematous papules and plaques scattered near the mandible and anterolateral neck

**Figure 2:**
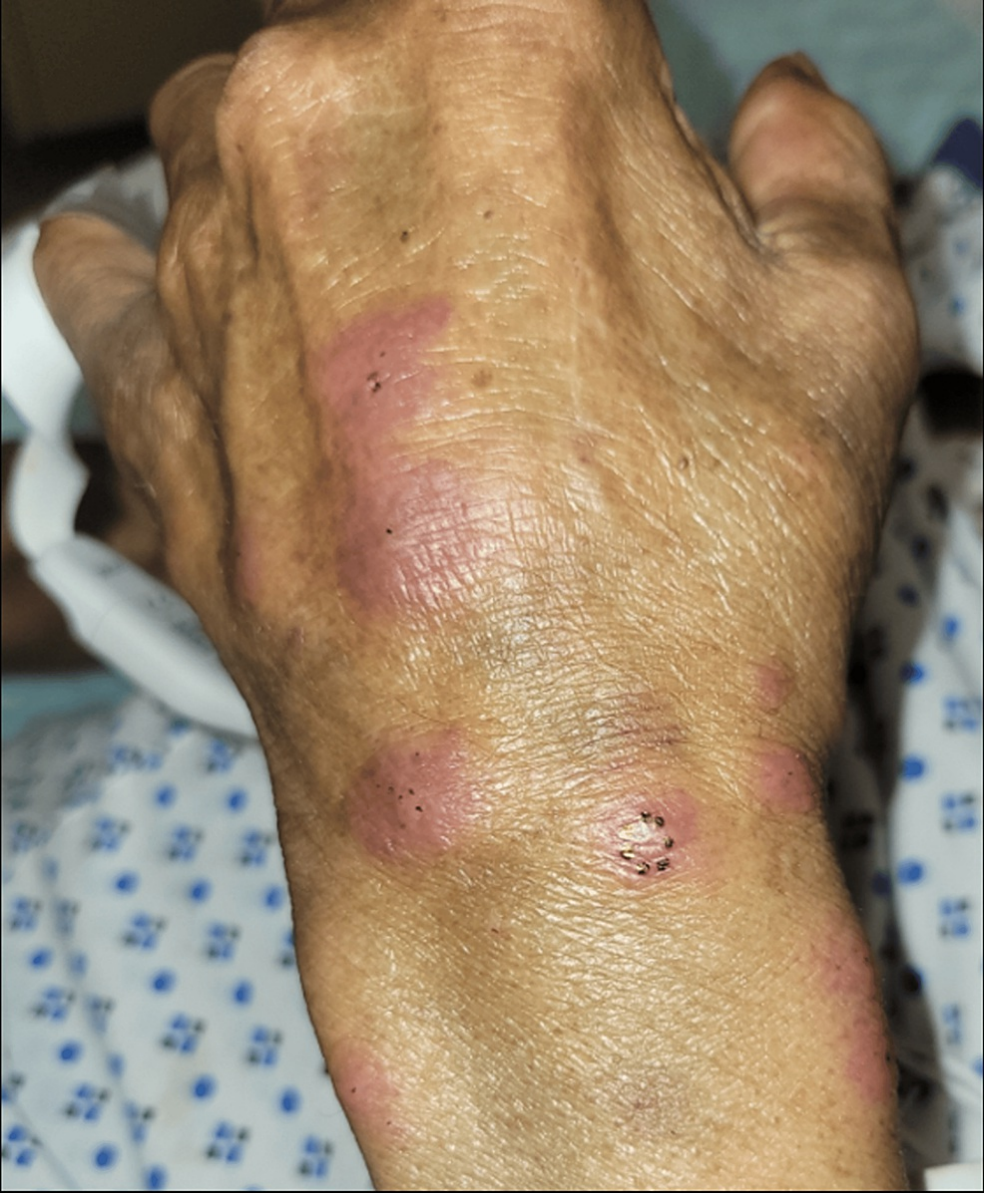
Erythematous, edematous plaques and nodules on the left hand and the left dorsal forearm

**Figure 3:**
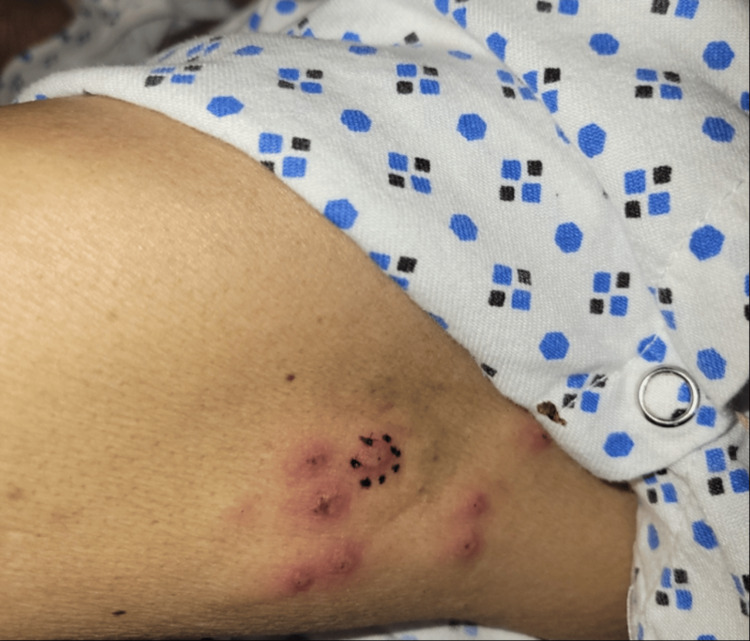
Erythematous papules and plaques on the left upper arm

Dermatology was consulted for a 67-year-old male with fever, migratory joint pains, and painful skin lesions in the intensive care unit. The patient’s past medical history included myelodysplastic syndrome (MDS), recently treated *Mycobacterium avium* complex (MAC) infection, latent tuberculosis (TB), coronary artery disease, type II diabetes, and chronic kidney disease. His MDS was initially treated with a brief course of erythropoietin. The patient was also undergoing assessment for an allogeneic stem cell transplant. Physical exam noted pink to violaceous, juicy, edematous papules, plaques, and nodules on the neck, upper arms, dorsal forearms, and hands (Figures 1-3).

**Figure 4:**
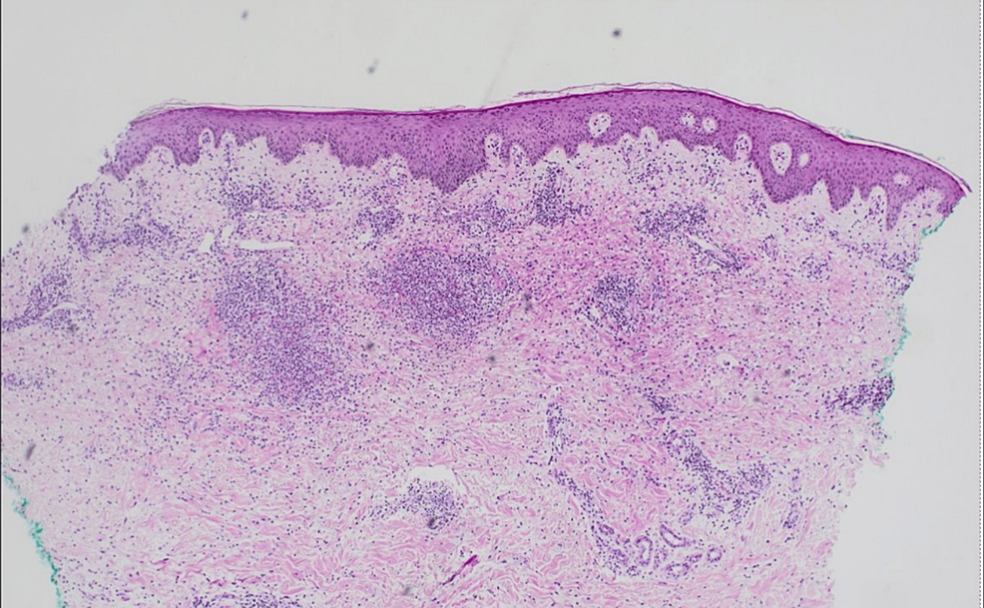
Significant interstitial and perivascular histiocytoid and lymphoid infiltrates seen in the dermis with edema present (hematoxylin and eosin stain 40x)

**Figure 5:**
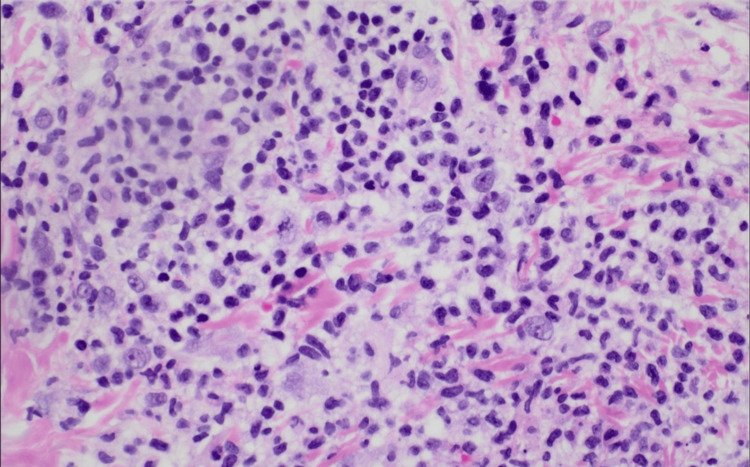
Scattered histiocytoid and lymphoid cells seen with prominent nucleoli and nuclear atypia; notably, there is a paucity of mature neutrophils in the inflammatory population (hematoxylin and eosin stain 400x)

Histopathological examination of punch biopsy of the left arm revealed a dense accumulation of dermal interstitial and perivascular histiocytoid and lymphocytic cells with sparse neutrophilic infiltrates along with papillary dermal edema (Figures 4, 5). Fungal, bacterial, and Epstein-Barr viral testing were negative. Immunohistochemistry and fluorescence in situ hybridization for trisomy 8 were also conducted to further rule out leukemia cutis and infiltration of neoplastic cells into the dermis, given the patient’s myelodysplasia. The distinctive combination of atypical histiocytoid and lymphocytic infiltrates on histology and the absence of positive microbiological testing, alongside the clinical presentation, strongly suggested histiocytoid Sweet’s syndrome.

Due to concern for the patient’s immunocompromised status, he was initially treated with dapsone and colchicine. However, the lack of clinical improvement necessitated the escalation of treatment to systemic corticosteroids. His latent TB was treated with rifampin while prednisone 1 mg/kg was initiated, leading to the discontinuation of dapsone and colchicine. After four days of prednisone treatment, the fevers subsided, and the patient was discharged. At outpatient follow-up, dapsone 50 mg and topical triamcinolone 0.1% cream were re-introduced to the regimen while the patient was tapered off prednisone. Resolution of skin lesions was observed. 

## Discussion

In this case, the primary challenge to treatment was the complex medical history of the patient. The patient’s history of latent tuberculosis compounded with his recently treated MAC infection created barriers to starting systemic corticosteroid treatment. Therefore, the patient was initially started on dapsone and colchicine, however, the patient had no clinical improvement. Following a thorough evaluation of the risks and benefits, a decision was made to start high-dose prednisone therapy concurrently with rifampin for latent tuberculosis. In immunocompromised patients, systemic corticosteroid therapy must be cautiously introduced, as it can exacerbate active infections or trigger latent disease. Starting rifampin alongside the prednisone allowed the latent manifestations of the patient’s tuberculosis to be resolved while providing a more effective treatment for his dermatologic condition. The literature indicates that patients suffering from Sweet’s syndrome, particularly those with underlying immunocompromising conditions, such as HIV, have successfully continued to use prednisone for the management of their dermatosis [4]. Patients with tuberculosis-associated Sweet’s syndrome have also received prednisone while also being actively treated for their infection with an adequate anti-tubercular drug regimen [5]. In patients with immunocompromised states, steroid-sparing therapies, such as indomethacin or colchicine, should be initially started, as they may adequately treat the lesions, however, providers should not refrain from using systemic steroids if the initial therapy fails.

While Sweet’s syndrome can be idiopathic, other conditions, such as infection or malignancy, typically precede the onset of this dermatosis. Interestingly, in patients with MDS, histiocytoid Sweet’s syndrome (H-SS) has been reported more frequently than the usual neutrophilic presentation (N-SS) [6]. The cutaneous manifestations of the disease remain similar in both variants, however, the histopathological characteristics are distinctive. It is suggested that the histiocytoid immature infiltrate in H-SS may be an earlier precursor to the N-SS histological appearance or that the inflammatory infiltrate seen in H-SS may instead be immature neutrophils [2]. In this case, MDS could potentially have triggered H-SS, as suggested by the atypical histopathological findings. H-SS may also serve as a prognostic and predictive indicator for the occurrence of MDS in patients [7]. According to a case series, H-SS has been noted to occur two to eight years before the onset of myelodysplasia indicating that the association between Sweet’s syndrome, specifically H-SS, and MDS remains strong [7]. While this patient had been diagnosed with MDS before Sweet’s syndrome, the literature supports that there is an indication that the etiology of his H-SS may have been from his MDS.

## Conclusions

In conclusion, this case represents an intricate presentation of Sweet’s syndrome characterized by histiocytoid and lymphocytic infiltrates, occurring in the setting of myelodysplasia and complicated by the patient's immunosuppressive status. While neutrophilic Sweet’s syndrome is the predominant variant in most cases, histiocytoid Sweet’s syndrome should be considered in patients with myelodysplasia. Steroid therapy may be a viable option to help resolve symptoms, however, clinicians must evaluate the benefits against the risk of additional immunosuppression. Individual patient factors and a history of previous treatments should also be assessed to optimize dermatologic treatment.
